# ADAMTS1 alters blood vessel morphology and TSP1 levels in LNCaP and LNCaP-19 prostate tumors

**DOI:** 10.1186/1471-2407-10-288

**Published:** 2010-06-14

**Authors:** Heléne Gustavsson, Tajana Tešan, Karin Jennbacken, Kouji Kuno, Jan-Erik Damber, Karin Welén

**Affiliations:** 1 Department of Urology, Lundberg Laboratory for Cancer Research, Institute of Clinical Sciences, Sahlgrenska Academy at University of Gothenburg, Gothenburg, Sweden; 2 Division of Molecular Oncology, Cancer Research Institute, Kanazawa University, Kanazawa, Japan

## Abstract

**Background:**

Decreased expression of the angiogenesis inhibitor ADAMTS1 (ADAM metallopeptidase with thrombospondin type 1 motif, 1) has previously been reported during prostate cancer progression. The aim of this study was to investigate the function of ADAMTS1 in prostate tumors.

**Methods:**

ADAMTS1 was downregulated by shRNA technology in the human prostate cancer cell line LNCaP (androgen-dependent), originally expressing ADAMTS1, and was upregulated by transfection in its subline LNCaP-19 (androgen-independent), expressing low levels of ADAMTS1. Cells were implanted subcutaneously in nude mice and tumor growth, microvessel density (MVD), blood vessel morphology, pericyte coverage and thrombospondin 1 (TSP1) were studied in the tumor xenografts.

**Results:**

Modified expression of ADAMTS1 resulted in altered blood vessel morphology in the tumors. Low expression levels of ADAMTS1 were associated with small diameter blood vessels both in LNCaP and LNCaP-19 tumors, while high levels of ADAMTS1 were associated with larger vessels. In addition, TSP1 levels in the tumor xenografts were inversely related to ADAMTS1 expression. MVD and pericyte coverage were not affected. Moreover, upregulation of ADAMTS1 inhibited tumor growth of LNCaP-19, as evidenced by delayed tumor establishment. In contrast, downregulation of ADAMTS1 in LNCaP resulted in reduced tumor growth rate.

**Conclusions:**

The present study demonstrates that ADAMTS1 is an important regulatory factor of angiogenesis and tumor growth in prostate tumors, where modified ADAMTS1 expression resulted in markedly changed blood vessel morphology, possibly related to altered TSP1 levels.

## Background

Extracellular matrix (ECM) proteases are involved in several steps of cancer development and progression, including angiogenesis and metastasis. By cleavage of ECM components, proteases regulate endothelial cell migration and the selective release and modulation of pro- and anti-angiogenic factors [1].

ADAMTS1 (ADAM metallopeptidase with thrombospondin type 1 motif, 1) is a widely expressed matrix metalloproteinase with documented roles in angiogenesis and tumor biology [2-6]. It has been described as a potent anti-angiogenic factor that effectively inhibits endothelial cell proliferation and angiogenesis in experimental assays [2]. As the name indicates, the ADAMTS1 protein comprises a metalloproteinase domain and three thrombospondin (TSP) type I motifs [7], both of which is important for the angioinhibitory capacity. The TSP type I motifs of ADAMTS1 have been reported to directly bind vascular endothelial growth factor (VEGF)_165_, and thereby block its angiogenic function [8]. In addition, the metalloproteinase domain has the ability to release anti-angiogenic fragments through cleavage of matrix-bound TSP1 and -2 [9]. TSP1 is one of the most studied endogenous inhibitor of angiogenesis, and downregulation of TSP1 is common in a variety of tumor types, including prostate cancer [10].

ADAMTS1 has been reported to efficiently inhibit tumor growth and metastasis in different experimental cancer models by blocking angiogenesis [3-5], and decreased expression of ADAMTS1 has been reported in human malignancies [11-13]. However, the involvement of ADAMTS1 in tumor progression is complex, with data also describing ADAMTS1 as a tumor promoting factor [4-6]. The tumor promoting effect is believed to involve the release of growth factors from ECM, and there are studies suggesting that the proteolytic status of ADAMTS1 is of importance for its effect on tumor growth [4,5].

In human prostate cancer, angiogenesis is related to clinical stage, progression, metastasis and survival [14-18]. In addition, androgen-independent or castration resistant prostate cancer (i.e. tumors relapsing from androgen deprivation therapy) displays higher MVD compared to androgen-dependent tumors [19-21]. Thus, factors affecting regulation of blood vessels and angiogenesis are of importance for the progression of prostate cancer, and may also be candidate targets for anti-angiogenic treatment. In a previous study, we identified ADAMTS1 as a gene that was downregulated when the androgen-dependent human prostate cancer cell line LNCaP progressed into an androgen-independent subline, LNCaP-19 [22]. This transition into androgen-independency was also associated with enhanced malignancy, increased MVD, altered blood vessel morphology and less pericyte covered vessels [23-25]. Furthermore, decreased expression of ADAMTS1 was found in tumor tissue from prostate cancer patients compared to benign prostate tissue, and low levels of ADAMTS1 were associated with increased MVD and metastasis in androgen-independent tumors [19].

This study was conducted to investigate the actual function of ADAMTS1 in prostate cancer. ADAMTS1 expression was downregulated in LNCaP cells (androgen-dependent) with shRNA technology and was upregulated in LNCaP-19 (androgen-independent) by transfection with an expression vector containing full-length ADAMTS1. We report that modified expression of ADAMTS1 resulted in markedly changed blood vessel morphology and TSP1 levels in the tumor xenografts, while MVD and pericyte coverage was unaffected. Moreover, the effect of ADAMTS1 on tumor growth was different in LNCaP and LNCaP-19. The results from this study demonstrate that ADAMTS1 is an important regulatory factor of angiogenesis and tumor growth in prostate tumors.

## Methods

### Cell lines and cell culture

The androgen-dependent human prostate cancer cell line LNCaP was obtained from American Type Culture Collection (ATCC, Manassas, VA). The androgen-independent cell line LNCaP-19 was previously established from LNCaP in our laboratory and cells were maintained as previously described [23].

### Expression vectors and transfection of cells

SureSilencing shRNA plasmids for human ADAMTS1 with neomycin (#KH01149N, SABiosciences, Frederick, MD) were used to downregulate the expression of ADAMTS1 by RNA interference in LNCaP cells. A plasmid expressing shRNA that does not match any human, mouse or rat gene was included as control. LNCaP-19 cells were transfected with a mammalian expression vector containing the full-length wild-type mouse ADAMTS1 [4] or empty control vector (pcDNA3). Cells were transfected over night with 8 μg DNA and 20 μl Lipofectamine 2000 (Invitrogen, Carlsbad, CA) in 6 ml culture medium without antibiotics. Three days after transfection, cells were subjected to selective pressure by adding Geneticin (400 μg/ml, Invitrogen). Three weeks later, stably transfected clones were isolated, expanded and screened for ADAMTS1 expression by real-time RT-PCR and western blotting. Several clones were obtained and one clone from each group was selected for *in vivo *studies.

### Animal experiments

Isolated clones and wild-type cells were implanted subcutaneously in male athymic BALB/c nude mice, 8-10 weeks old (Charles River Laboratories, Wilmington, MA). The use of animals was approved by the animal ethical committee in Gothenburg (Nr: 228-2006). Two million cells, diluted in 100 μl culture medium and 100 μl Matrigel (BD Biosciences, Bedford, MA), were injected subcutaneously in the flank of the mice. To represent the clinical situation, LNCaP cells (wild-type = L wt, control transfected = LC and ADAMTS1 shRNA-transfected = LA-) were implanted in intact mice, while LNCaP-19 cells (wild-type = L19 wt, control transfected = L19C and ADAMTS1 transfected = L19A+) were implanted in castrated mice. Each clone was implanted in eight mice. Mice used for implantation of LNCaP-19 clones were castrated under anesthesia three days before inoculation. In a separate control experiment L19C and L19A + were also implanted in intact mice. Tumors were measured with a caliper and tumor volumes were calculated using the formula; (length × width^2^)/2. When tumor size reached about 1.3 cm^3^, or after a maximum of 15 weeks, mice were sacrificed and tumors were removed and weighed. Tumor take rate was defined as percentage of animals with a palpable tumor at different time points after implantation. Tumor growth rate was calculated as tumor weight (mg) divided with the number of days from implantation to sacrifice, and then related to the growth rate of wt tumors. One part of the tumors was fixed in formalin for paraffin embedding and one part was frozen in liquid nitrogen and stored in -80°C.

### RNA preparation and real-time RT-PCR

Total RNA from cells and tumor tissue was isolated with the RNeasy Plus Mini kit and treated with RNase-free DNase I (Qiagen, Hilden, Germany) according to the manufacturer's protocol. RNA concentration and purity were determined spectrophotometrically, and the integrity of RNA was confirmed by gel electrophoresis. Reverse transcription of total RNA was performed as previously described [22]. Real-time RT-PCR was performed with TaqMan Gene Expression Assays in a 7500 Fast Real-Time PCR System (Applied Biosystems, Foster City, CA), according to the manufacturer's protocol. The TaqMan Gene Expression Assays used were; human ADAMTS1 (Hs00199608_m1), mouse Adamts1 (Mm00477355_m1) and 18S (Hs99999901_s1). The real-time RT-PCR analysis was performed in duplicates and was repeated twice. Data were analyzed according to the ΔΔCt method for relative quantification according to Applied Biosystems' instructions and were normalized to the expression of 18S rRNA.

### Protein preparation and Western blotting

Protein extraction and western blotting was performed as previously described [22]. Primary antibodies used were against; human ADAMTS1 (1:500, #A4476, Sigma-Aldrich, St Louis, MO), mouse ADAMTS1 (1:5000, kindly provided by Prof. Joanne S. Richards [26]), and TSP1 (1:400, #MS-421-P1, NeoMarkers, Fremont, CA). The TSP-1 antibody reacts with the C-terminal of the protein and has earlier been reported to detect full-length TSP1 and ADAMTS1 cleaved TSP1 [9]. As loading control an antibody against actin (1:500, #A2066, Sigma-Aldrich) was used. Chemiluminescent signals were visualized by the LAS-4000 CCD camera (Fujifilm, Tokyo, Japan) and semi-quantitative analysis of immunoreactive bands was performed with the free computer software ImageJ http://rsb.info.nih.gov/ij/.

### Cell proliferation assay

Cells were seeded in triplicates in 96-well plates at a density of 5000 cells/well in regular culture medium. Cells were incubated for four days and then frozen at -80°C. Proliferation was analyzed using the CyQUANT Cell Proliferation Assay Kit (Invitrogen) according to the manufacturer's protocol. A fluorescent dye that binds to DNA was added and fluorescence was measured with a Victor^3 ^multilabel plate reader (PerkinElmer, Waltham, MA). Proliferation experiments were repeated twice.

### Detection of blood vessels in tumors

MVD, blood vessel morphology and pericyte coverage were analyzed by immunohistochemistry. For determination of MVD and blood vessel morphology, sections were stained with an antibody against CD34 (1:20, #ab8158, Abcam, Cambridge, UK). To analyze pericyte coverage, another set of sections were double stained with the CD34 antibody and an antibody against α-SMA (1:20, #A5691, Sigma-Aldrich). Immunohistochemistry was performed as previously described [22,25]. MVD was evaluated by counting the number of CD34-positive vessels in five objective fields of representative areas in each section. Blood vessel morphology was assessed by grouping the tumors into two groups according to the form and distribution of vessels. Tumors characterized by large, thick and singly distributed vessels, often with a visible lumen were denoted as LV (large vessels), while tumors characterized by small and thin vessels forming networks without a visible lumen were assigned as SV (small vessels). The evaluation was based on the total area of the section and included both peripheral and central parts of the tumor. To evaluate pericyte coverage index, all CD34-positive vessels associated with α-SMA positive cells were counted and divided with the total number of CD34-positive vessels in five objective fields in each section. All analyses were performed independently by two investigators at 200 × magnification.

### Statistical analyses

Data are presented as mean ± SEM. Mann Whitney U test was used to compare differences between groups. Fisher's exact test was used to compare the tumor take rate between groups. A *P*-value < 0.05 was considered statistically significant. All statistical analyses were performed using SPSS 16.0 software for Windows.

## Results

### Modified expression of ADAMTS1 in LNCaP and LNCaP-19 cells

**Figure 1:**
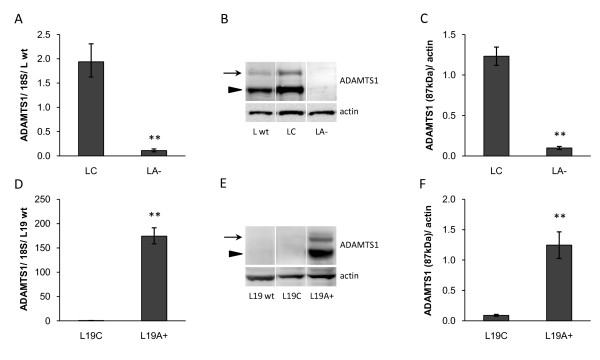
**Expression of ADAMTS1 in tumor xenografts**. (**A, D**) mRNA expression of ADAMTS1 in tumor xenografts was analyzed by real-time RT-PCR, normalized to the expression of 18S rRNA and presented as relative expression compared to wild-type tumors. (**B, E**) Protein levels of ADAMTS1 in tumor xenografts were analyzed by western blotting. ADAMTS1 was detected in its latent form (110 kDa, arrows) as well as in its active form (87 kDa, arrowheads) and actin was used as loading control. Western blotting membranes showing one representative sample from each group. (**C, F**) Densitometric analysis presented as relative levels of active ADAMTS1 (87 kDa) to actin. Results are presented as mean ± SEM. Mann Whitney U test was used to compare differences between groups, ** = *P *< 0.01. (**A-C**) LNCaP wild-type (L wt, n = 8), control transfected LNCaP (LC, n = 8) and ADAMTS1 shRNA-transfected LNCaP (LA-, n = 6). (**D-F**) LNCaP-19 wild-type (L19 wt, n = 6), control transfected LNCaP-19 (L19C, n = 8) and ADAMTS1 transfected LNCaP-19 (L19A+, n = 6).

LNCaP cells (L wt) that originally express significant amounts of ADAMTS1 were stably transfected with shRNA plasmids against ADAMTS1 (LA-) or control vector (LC). LNCaP-19 cells (L19 wt), originally expressing low amounts of ADAMTS1, were stably transfected with an expression vector containing the full-length ADAMTS1 (L19A+) or control vector (L19C). To represent the clinical situation LNCaP cells were inoculated in intact mice, while LNCaP-19 cells were implanted in castrated mice. Real-time RT-PCR and western blotting analysis revealed significantly decreased mRNA and protein levels of ADAMTS1 in LA- tumors compared to LC tumors (Figure 1A, B, C) and an obvious increase in ADAMTS1 expression in L19A + tumors compared to L19C tumors (Figure 1D, E, F). Both the latent form (110 kDa) and the active form (87 kDa) of ADAMTS1 protein that are present in LNCaP were also present in L19A + tumors.

### Tumor growth of LNCaP and LNCaP-19 was differently affected by ADAMTS1

**Figure 2:**
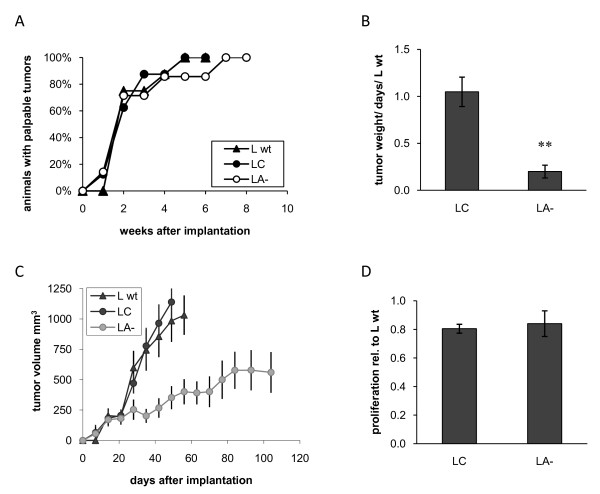
**Growth of LNCaP clones *in vivo *and *in vitro***. LNCaP cells were implanted subcutaneously in nude mice and the experiment was ended when tumor size reached about 1.3 cm^3^, or after a maximum of 15 weeks. LNCaP wild-type (L wt, n = 8), control transfected LNCaP (LC, n = 8) and ADAMTS1 shRNA-transfected LNCaP (LA-, n = 7). (**A**) Tumor take rate is presented as percentage of animals with a palpable tumor at different time points after implantation. Fisher's exact test was used to compare the tumor take rate between groups. (**B**) Tumor growth rate was calculated as tumor weight (mg) divided with the number of days from implantation to sacrifice and presented as the relative growth rate compared to wild-type tumors. Results are presented as mean ± SEM. Mann Whitney U test was used to compare differences between groups, ** = *P *< 0.01. (**C**) Tumor volumes were calculated as (length × width^2^)/2. (**D**) Proliferation rate *in vitro *of LC and LA- in relation to L wt. Cells were seeded in 96-well plates in their regular cell culture medium. Four days later, DNA was quantified by a fluorescent dye and values were related to the wild-type cells. Mean ± SEM of two independent experiments with triplicates are shown.

**Figure 3:**
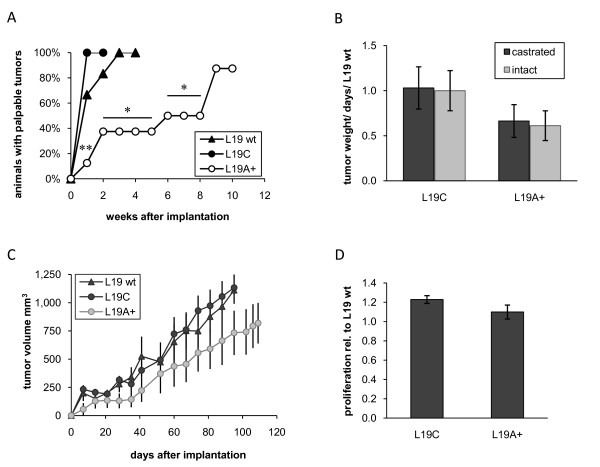
**Growth of LNCaP-19 clones *in vivo *and *in vitro***. LNCaP-19 cells were implanted subcutaneously in nude mice and the experiment was ended when tumor size reached about 1.3 cm^3^, or after a maximum of 15 weeks. LNCaP-19 wild-type (L19 wt, n = 6), control transfected LNCaP-19 (L19C, n = 8) and ADAMTS1 transfected LNCaP-19 (L19A+, n = 8) (**A**) Tumor take rate is presented as percentage of animals with a palpable tumor at different time points after implantation. Fisher's exact test was used to compare the tumor take rate between groups, ** = *P *< 0.01, * = *P *< 0.05 L19A+ vs. L19C. (**B**) Tumor growth rate of L19C and L19A+ in castrated as well as in intact mice (intact mice; L19C, n = 8 and L19A+, n = 7) was calculated as tumor weight (mg) divided with the number of days from implantation to sacrifice and presented as the relative growth rate compared to wild-type tumors. Results are presented as mean ± SEM. Mann Whitney U test was used to compare differences between groups. (**C**) Tumor volumes were calculated as (length × width^2^)/2. (**D**) Proliferation rate *in vitro *of L19C and L19A+ in relation to L19 wt. Cells were seeded in 96-well plates in their regular cell culture medium. Four days later, DNA was quantified by a fluorescent dye and values were related to the wild-type cells. Mean ± SEM of two independent experiments with triplicates are shown.

Decreased levels of ADAMTS1 did not affect tumor take of androgen-dependent LNCaP cells (Figure 2A), but strongly inhibited tumor growth rate. Tumor growth rate, measured as tumor weight/days in relation to LNCaP wt, was reduced with approximately 80% from 1.05 (±0.16) in LC tumors to 0.20 (±0.07) in LA- tumors (*P *< 0.01) (Figure 2B). Measurements of tumor volumes displayed similar results (Figure 2C). However, the proliferation rate *in vitro *was not affected (Figure 2D). In contrast, increased levels of ADAMTS1 in LNCaP-19 resulted in markedly slower tumor establishment. All mice in the L19C group had palpable tumors already after one week, while tumors in the L19A + group gradually became palpable during 9 weeks (Figure 3A). The relative tumor growth rate was 1.03 (±0.23) in L19C tumors and 0.66 (±0.18) in L19A + tumors (*P *= 0.279) (Figure 3B). Similar results were obtained when measuring tumor volumes (Figure 3C). However, there was no statistically significant effect on tumor growth rate once tumors were established. No difference in proliferation rate was observed *in vitro *(Figure 3D).

The discrepancy in tumor growth regulation between LNCaP and LNCaP-19 was not due to the fact that LNCaP-19 tumors were growing in castrated mice, while LNCaP tumors were growing in intact mice, since similar results were obtained when L19C and L19A + cells were implanted in intact mice as control (Figure 3B).

### ADAMTS1 altered tumor blood vessel morphology but not MVD or pericyte coverage

**Figure 4:**
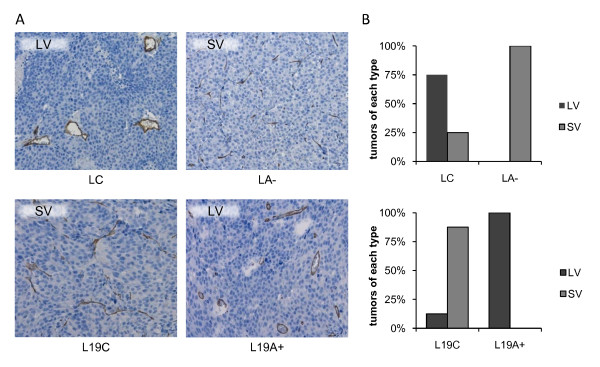
**Blood vessel morphology in tumor xenografts**. Blood vessels were stained for CD34 and morphology was evaluated in light microscopy at 200 × magnification. Tumors characterized by large and thick, singly distributed vessels, often with a visible lumen were assigned as LV = large vessels, while tumors characterized by small and thin vessels forming networks without a visible lumen were assigned as SV = small vessels. (**A**) Images showing the blood vessel morphology in typical LV and SV tumors in LNCaP and LNCaP-19 tumors with modified ADAMTS1. (**B**) Tumors were classified unaware of origin into LV or SV. Histograms show the distribution of tumors between LV and SV in LNCaP tumors (upper panel); control transfected LNCaP (LC, n = 8) and shRNA transfected LNCaP (LA-, n = 6) and in LNCaP-19 tumors (lower panel); control transfected LNCaP-19 (L19C, n = 8) and ADAMTS1 transfected LNCaP-19 (L19A+, n = 7).

**Figure 5:**
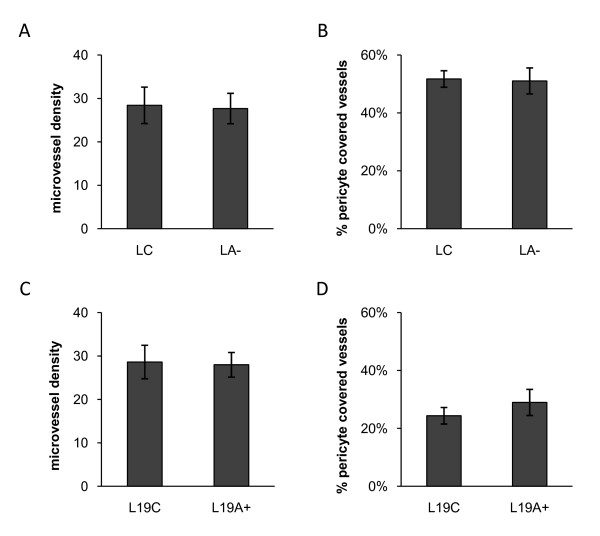
**Microvessel density and pericyte covered blood vessels in tumor xenografts**. (**A, C**) Microvessel density. Blood vessels were stained for CD34 and the number of vessels was counted in five fields of each tumor in light microscopy at 200 × magnification. Results are presented as mean number of vessels per field ± SEM. (**B, D**) Percentage of pericyte covered blood vessels. Blood vessels were double stained for CD34 and α-SMA. The number of α-SMA positive blood vessels were counted and divided with the total number of CD34 positive vessels in five fields of each tumor at 200 × magnification. Results are presented as mean ± SEM. (**A-B**) Control transfected LNCaP (LC, n = 8) and shRNA-transfected LNCaP (LA-, n = 6). (**C-D**) Control transfected LNCaP-19 (L19C, n = 8) and ADAMTS1 transfected LNCaP-19 (L19A+, n = 7).

Modified expression of ADAMTS1 induced a clear difference in blood vessel morphology. In LNCaP tumors, blood vessels in the L wt and LC groups were observed as singly distributed vessels that were larger, thicker and rounder in shape often with a visible lumen (assigned as LV = large vessels). In contrast, when ADAMTS1 was downregulated (LA-), the tumors were dominated by small and thin vessels forming long networks, mainly without a visible lumen (assigned as SV = small vessels). The blood vessel morphology was similarly affected by ADAMTS1 expression in LNCaP-19 tumors. The tumors with low ADAMTS1 (L19 wt and L19C) were dominated by smaller and thinner vessel networks (SV), while L19A + tumors displayed singly distributed blood vessels that were rounder and thicker in shape (LV) (Figure 4). However, ADAMTS1 did not affect MVD (Figure 5A, C) or the proportion of α-SMA positive vessels, representing pericyte covered vessels (Figure 5B, D), neither in LNCaP nor in LNCaP-19 tumors.

### ADAMTS1 affected the TSP1 levels in tumors

**Figure 6:**
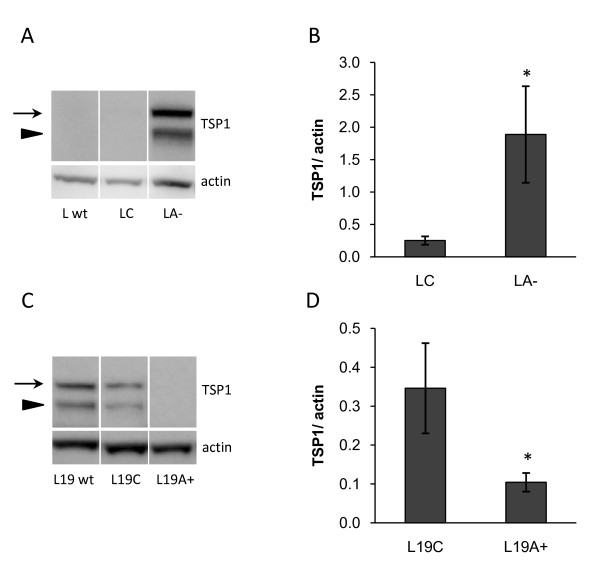
**TSP1 levels in tumor xenografts**. (**A, C**) TSP1 levels in tumor xenografts were analyzed by western blotting. TSP1 was detected in its full-length form (145 kDa, arrows) as well as in a smaller cleaved form (110 kDa, arrowheads). Actin was used as loading control. Western blotting membranes showing one representative sample from each group. (**B, D**) Densitometric analysis presented as relative levels of TSP1 to actin. Results are presented as mean ± SEM. Mann Whitney U test was used to compare differences between groups, * = P < 0.05. (**A-B**) LNCaP tumors from LNCaP wild-type (L wt, n = 8), control transfected cells (LC, n = 8) and ADAMTS1 shRNA-transfected cells (LA-, n = 6). (**C-D**) LNCaP-19 tumors from LNCaP-19 wild-type (L19 wt, n = 6), control transfected cells (L19C, n = 8) and ADAMTS1 transfected cells (L19A+, n = 6).

Since ADAMTS1 has been reported to cleave TSP1, we analyzed the protein forms of TSP1 by western blotting. TSP1 was detected as two immunoreactive bands of approximately the same size that previously was described as full-length TSP1 (145 kDa) and ADAMTS1 cleaved TSP1 (110 kDa) [9]. However, modified expression of ADAMTS1 did not alter the ratio between the two detected protein forms either in LNCaP or LNCaP-19 tumors. Instead, the total protein levels of detected TSP1 were markedly affected by ADAMTS1 levels. TSP1 was present in L19 wt, but not in L wt. Downregulation of ADAMTS1 in LNCaP resulted in increased levels of TSP1 (Figure 6A, B), and upregulation of ADAMTS1 in LNCaP-19 was associated with decreased levels of TSP1 (Figure 6C, D).

## Discussion

In the present study, we modified the expression of ADAMTS1 in an experimental model of prostate cancer to investigate the function of this protein. The main findings were that ADAMTS1 altered the blood vessel morphology and TSP1 levels in the tumor xenografts. In addition, the tumor growth was differently affected by ADAMTS1 in androgen-dependent LNCaP and androgen-independent LNCaP-19 tumors.

In prostate cancer, an increased angiogenesis is observed in androgen-independent and metastatic disease and this is probably important for the rapid growth and spreading of the cancer [17,19,23]. Since ADAMTS1 is known as a potent inhibitor of angiogenesis in other systems and previously has been reported to decrease during prostate cancer progression, we investigated its effects on tumor blood vessels. Interestingly, we found that modified expression of ADAMTS1 markedly altered the blood vessel morphology in these tumor xenografts, while the number of blood vessels was unaffected. During the transition of LNCaP into LNCaP-19, an altered blood vessel morphology was observed together with increased MVD and the loss of ADAMTS1 [22,23]. In this study, downregulation of ADAMTS1 in LNCaP resulted in a blood vessel phenotype characteristic of LNCaP-19 tumors with small diameter vessels forming long networks. By upregulation of ADAMTS1 in LNCaP-19, the vessel morphology could be reversed into a phenotype resembling the blood vessels in LNCaP tumors, characterized by larger vessels. These results demonstrate that ADAMTS1 is a regulator of blood vessel biology also in prostate tumors. The significance of this switch in blood vessel morphology for tumor progression is not extensively investigated. However, it was recently shown in a large prospective study of prostate cancer patients that the vessel morphology is of importance for the clinical outcome. It was shown that small blood vessels were associated with poorly differentiated tumors. More interestingly, men whose tumors had a small vessel diameter or a small vessel area were about 6 times more likely to develop lethal prostate cancer, even after adjustment for age, differentiation (Gleason score) and PSA [27]. This new data together with our results presented here, implies that loss of ADAMTS1 could be an important mediator of the progression into more aggressive prostate cancer.

ADAMTS1 has previously been reported to regulate angiogenesis by cleaving TSP1, which results in the release of TSP1 from ECM and thereby to decreased angiogenesis [9]. However, modified ADAMTS1 expression did not affect the cleavage of TSP1 in LNCaP or LNCaP-19 tumors. Instead, we found that ADAMTS1 expression was inversely related to the total levels of TSP1 in the tumor xenografts. Since TSP1 is a potent angiogenesis inhibitor, this may explain why the MVD in the tumor xenografts was not affected by altered levels of ADAMTS1. Interestingly, TSP1 has also been described to affect blood vessel morphology. Mammary tumors in TSP1-null mice have been reported to display significantly larger blood vessels than tumors in wild-type mice [28,29]. This is in accordance with our study, where decreased expression of ADAMTS1 was associated with increased TSP1 and a switch from large vessels into small vessels, while overexpression of ADAMTS1 resulted in loss of TSP1 and a switch back to larger vessels. Thus, the ADAMTS1-mediated effect on TSP1 levels could be a possible mechanism responsible for the switch in blood vessel morphology observed.

Tumor blood vessels are often characterized by decreased pericyte coverage, making them less stabilized, more permeable and more sensitive to angiogenic stimuli [30]. LNCaP tumors have previously been described to have more vessels stabilized by pericytes than LNCaP-19 tumors [25]. However, modified expression of ADAMTS1 did not influence the degree of pericyte coverage, indicating that altered vessel stabilization by pericytes is not involved in the altered blood vessel morphology observed.

ADAMTS1 is a multifunctional protein reported to have both pro- and anti-tumorigenic properties. Transition of the cell line LNCaP into its more malignant subline LNCaP-19 has previously been reported to result in loss of ADAMTS1 together with a more rapid tumor establishment [22,23]. Also in the human prostate, ADAMTS1 was downregulated in tumor tissue compared to benign tissue and decreased expression of ADAMTS1 was associated with increased angiogenesis and metastasis in androgen-independent tumors [19]. In this study, re-expression of ADAMTS1 in LNCaP-19 greatly delayed tumor establishment, indicating that ADAMTS1 actually might function as a tumor suppressor of androgen-independent prostate tumors. However, once tumors were established the LNCaP-19 cells seem to escape from this inhibitory effect of ADAMTS1, since no obvious difference in tumor growth rate was observed. That increased expression of ADAMTS1 is associated with a less malignant phenotype of androgen-independent prostate tumors has also been shown in PC-3 tumors [31]. However, downregulation of ADAMTS1 in LNCaP resulted in a reduced tumor growth rate, suggesting that ADAMTS1 rather is a tumor promoting factor in androgen-dependent LNCaP tumors. Other experimental studies also report conflicting data regarding ADAMTS1 as a pro- or anti-tumorigenic factor, and the proteolytic status of ADAMTS1 has been proposed to be of importance for its effect [4,5]. However, in this study ADAMTS1 was only detected in its latent and full-length active form in both LNCaP and LNCaP-19 tumors, suggesting that this is not the reason to the difference in tumor growth regulation in this model. Since ADAMTS1 is a multifunctional protein mainly acting indirectly on tumor cells by regulating the availability and activity of factors present in the ECM, the constitution of the surrounding ECM is probably of great importance for the net effect of ADAMTS1.

## Conclusions

In conclusion, modified ADAMTS1 expression resulted in markedly changed blood vessel morphology and altered TSP1 levels in the tumors. Loss of ADAMTS1 was associated with small diameter vessels that have been associated with more aggressive prostate tumors. Altogether, the results presented in this paper further support that ADAMTS1 is an important regulatory factor of tumor growth and angiogenesis during prostate cancer progression.

## Competing interests

The authors declare that they have no competing interests.

## Authors' contributions

HG designed the study, performed the experiments, analyzed the data and drafted the manuscript. TT and KJ helped with the experiments and participated in revising the manuscript. KK established the ADAMTS1 expression vector system. JED and KW participated in the design of the study and analysis of data, drafted and revised the manuscript. All authors read and approved the final manuscript.

## Pre-publication history

The pre-publication history for this paper can be accessed here:

http://www.biomedcentral.com/1471-2407/10/288/prepub
